# Lesser-Known Cyanotoxins: A Comprehensive Review of Their Health and Environmental Impacts

**DOI:** 10.3390/toxins16120551

**Published:** 2024-12-19

**Authors:** Molham Al Haffar, Ziad Fajloun, Sami Azar, Jean-Marc Sabatier, Ziad Abi Khattar

**Affiliations:** 1 Faculty of Medicine and Medical Sciences, University of Balamand, Kalhat, Tripoli P.O. Box 100, Lebanon; molham.alhaffar@std.balamand.edu.lb (M.A.H.); sami.azar@balamand.edu.lb (S.A.); 2 Department of Biology, Faculty of Sciences 3, Lebanese University, Campus Michel Slayman Ras Maska, Tripoli 1352, Lebanon; ziad.fajloun@ul.edu.lb; 3 Laboratory of Applied Biotechnology (LBA3B), Department of Cell Culture, Azm Center for Research in Biotechnology and Its Applications, EDST, Lebanese University, Tripoli 1300, Lebanon; 4 CNRS, INP, Inst Neurophysiopathol, Aix-Marseille Université, 13385 Marseille, France

**Keywords:** cyanotoxins, cyanobacteria, neurotoxicity, dermatotoxicity, hepatotoxicity, harmful algal blooms, amyotrophic lateral sclerosis/parkinsonism–dementia complex (ALS-PDC), beta-methylamino-L-alanine (BMAA), aerosolized cyanobacteria

## Abstract

Cyanobacteria, also known as blue-green algae, are a diverse phylum of photosynthetic, Gram-negative bacteria and one of the largest microbial taxa. These organisms produce cyanotoxins, which are secondary metabolites that can have significant impacts on both human health and the environment. While toxins like Microcystins and Cylindrospermopsins are well-documented and have been extensively studied, other cyanotoxins, including those produced by *Lyngbya* and *Nostoc*, remain underexplored. These lesser-known toxins can cause various health issues in humans, including neurotoxicity, hepatotoxicity, and dermatotoxicity, each through distinct mechanisms. Moreover, recent studies have shown that cyanobacteria can be aerosolized and transmitted through the air over long distances, providing an additional route for human exposure to their harmful effects. However, it remains an area that requires much more investigation to accurately assess the health risks and develop appropriate public health guidelines. In addition to direct exposure to toxins, cyanobacteria can lead to harmful algal blooms, which pose further risks to human and wildlife health, and are a global concern. There is limited knowledge about these lesser-known cyanotoxins, highlighting the need for further research to understand their clinical manifestations and improve society’s preparedness for the associated health risks. This work aims to review the existing literature on these underexplored cyanotoxins, which are associated with human intoxication, elucidate their clinical relevance, address significant challenges in cyanobacterial research, and provide guidance on mitigating their adverse effects.

## 1. Introduction

Cyanobacteria, a diverse phylum of autotrophic microorganisms, are found in almost all environments on Earth [1]. These organisms, often referred to as blue-green algae, are capable of producing chlorophyll *a*, the primary pigment responsible for harvesting light energy and converting it into chemical energy through photosynthesis [2]. Additionally, cyanobacteria produce a pigment called phycocyanin, which gives them their characteristic bluish-green color when present in high concentrations. For this reason, they are commonly known as “blue-green algae” [1].

While many cyanobacteria play a crucial role in maintaining ecosystem balance and serve as a rich source of bioactive secondary metabolites, including antimicrobial agents [3], such as antimicrobial peptides, which are used to combat multidrug resistant microbes [4], a significant number of species produce toxic secondary metabolites, known as cyanotoxins [5]. These toxins are byproducts of several toxic cyanobacterial species that thrive in various environments, including freshwater, marine, and brackish systems. When conditions are favorable, cyanobacteria can proliferate rapidly, forming harmful algal blooms (HABs) that have increasingly become a global threat to ecosystems and public health [6,7,8,9]. The frequency and intensity of cyanobacterial blooms have increased in recent years, driven by human activities, such as agricultural runoff and wastewater discharge, as well as climate change. The influx of nutrients, such as nitrogen and phosphorus, into aquatic environments further accelerates cyanobacterial growth, promoting the production of large quantities of cyanotoxins [5,10,11,12,13,14].

The relationship between cyanobacteria and their environment is complex, with the growth and proliferation of these organisms being influenced by several factors. Optimal conditions for cyanobacterial growth include sufficient sunlight, appropriate pH levels, salinity, carbon availability, and high concentrations of nutrients [5]. When these conditions align, cyanobacteria can dominate aquatic systems, sometimes leading to large-scale toxic blooms [15].

**Figure 1:**
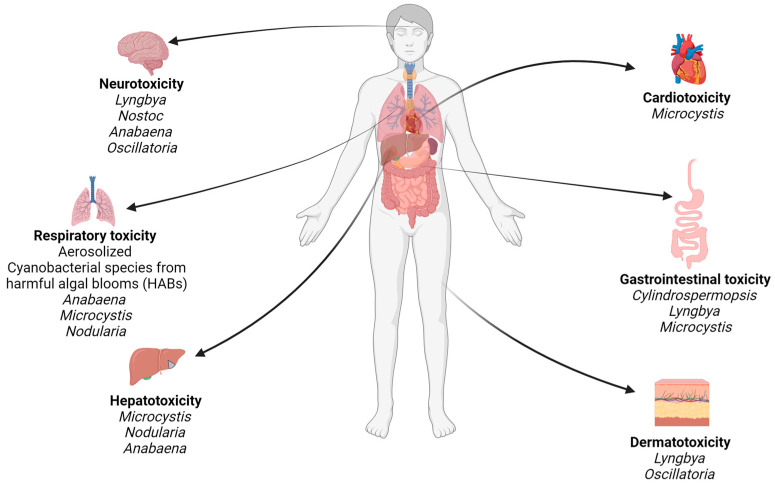
Toxic effects of various cyanobacteria on humans.

Cyanotoxins pose significant risks to human health (Figure 1) and ecosystems [16]. These toxins can enter water bodies and contaminate drinking water supplies or exert toxic effects on aquatic organisms, such as fish and shellfish [17], which can subsequently be consumed by humans [17]. Additionally, cyanotoxins can infiltrate irrigation systems and contaminate crops, providing another pathway for human exposure to these toxins [18,19,20,21,22]. Human exposure to cyanotoxins can happen through inhalation, ingestion, and direct skin contact; however, human exposure is most commonly linked to the consumption of toxin-laden drinking water or seafood [23]. Other exposure routes exist, such as cyanotoxins seeping into the environment through aerosolization, particularly in recreational settings near contaminated water bodies [24].

Despite their harmful effects, some cyanobacterial species offer substantial benefits and can be harnessed to address environmental and agricultural challenges [25]. Cyanobacteria play important roles in sustainable food production, enhancing soil fertility, and even contributing to the development of biofuels [26]. These beneficial applications are largely due to cyanobacteria’s ability to perform oxygenic photosynthesis, their environmental resilience, and their capacity to fix atmospheric nitrogen into bioavailable forms. As a result, certain species of cyanobacteria hold great promise in terms of agricultural and industrial use [27].

Cyanotoxins exhibit a wide range of physicochemical properties and toxicological profiles [28]. These toxins can be classified by their chemical structure, such as cyclic peptides, alkaloids, or lipopolysaccharides (LPS), or by the specific cyanobacterial species that produce them [28,29,30]. Each toxin also has a unique mechanism of action, affecting different organ systems and cellular pathways. For instance, some cyanotoxins act as hepatotoxins, while others are neurotoxins, interfering with neural signaling, and still others are dermatotoxins, causing skin irritation and inflammation [28].

The ingestion of cyanotoxins can result in a wide range of symptoms, with the most commonly reported being nausea, fever, and abdominal pain [28]. In contrast, skin exposure can also lead to symptoms like rashes, irritation, and dermatitis [16]. On the other hand, the inhalation of aerosolized cyanotoxins can result in respiratory distress [23]. Many cyanotoxins from well-known genera, such as *Microcystis*, *Anabaena*, and *Nodularia*, have been extensively studied and documented [31,32,33]. The metabolic products of cyanobacteria can have adverse effects on human health, causing diseases that can be life threatening and can even culminate in death [34]. Common hepatotoxins include microcystins (MCs) and cylindrospermopsins (CYNs), while anatoxins and saxitoxins (SXTs) are common neurotoxins. Lipopolysaccharides and lyngbyatoxin are dermatotoxins that can cause intoxication, along with other toxins (Table 1) [8,29,34]. MCs are commonly found in freshwater bodies, causing hepatotoxicity, and can also affect certain organs, including the kidneys, the GI tract, the lungs, and the heart, primarily through the consumption of contaminated drinking water and irrigated plants [29,35,36,37,38,39,40]. Anatoxins, potent neurotoxins primarily produced by the *Dolichospermum* species, irreversibly bind to acetylcholine receptors, overstimulating them, which leads to muscle spasms, breathing difficulties, convulsions, and potentially fatal respiratory failure if untreated [13,18], posing significant health risks to both humans and animals exposed to contaminated water. CYNs, produced by cyanobacteria, such as *Cylindrospermopsis raciborskii*, are stable, water-soluble toxins found in both freshwater and salty water, which can bioaccumulate in aquatic organisms and cause hepatotoxicity, neurotoxicity, and immunotoxicity, by inhibiting vital cellular processes, thereby posing risks to food safety and human health [41,42,43,44,45]. Similarly, STX, a potent neurotoxic alkaloid, inhibits voltage-gated sodium channels, causing paralysis, and bioaccumulates in aquatic organisms, like fish and shellfish, presenting a major public health concern, with symptoms ranging from mild to potentially fatal respiratory paralysis [46,47,48,49]. Given the significant risks posed by these well-known cyanotoxins, there is growing recognition of the need to better understand lesser-studied cyanotoxins, which may present similar or an even greater threat to human health, yet remain poorly researched in terms of their occurrence, toxicity, and mechanisms of action.

This work aims to review the existing literature on underexplored cyanotoxins, with a focus on their potential impact on human health. We will delve into the specific mechanisms by which these toxins exert their toxic effects, examine their environmental persistence, and discuss their significance as emerging public health threats. Additionally, we will explore methods for mitigating and preventing exposure to these toxins, providing recommendations on how to address the risks in both environmental and clinical contexts. By expanding our understanding of these lesser-known cyanotoxins, we will contribute to the development of more effective public health strategies and environmental management practices aimed at reducing the risks associated with cyanobacterial toxins.

## 2. Less Studied Cyanobacteria and Their Effects on Human Health and the Environment

While well-studied cyanotoxins, such as MCs and CYNs, have been shown to elicit significant harmful effects to both human health and the environment, other less commonly encountered cyanotoxins have been shown to trigger similar harmful effects that are just as significant and, in some cases, are more severe. In this section, we provide an overview of such cyanotoxins and information about the producing species, the health-related manifestations, and how best to manage and prevent calamities.

### 2.1. Debromoaplysiatoxin, Aplysiatoxin, and Lyngbyatoxin A of Moorea Producens

A common adverse effect of lyngbyatoxins, aplysiatoxin (AT), and debromoaplysiatoxin (DAT) in humans is skin irritation, termed “swimmer’s itch” [64]. All three are potent protein kinase C (PKC) activators and belong to a group of polyether toxins [65]. These toxins are mainly produced by *Lyngbya* species and have similar structural characteristics, including a polycyclic ring system. *Moorea producens* (formerly classified as *Lyngbya majuscula*) is a filamentous marine cyanobacterium that grows in deep, oceanside waters and poses health risks to humans and the environment [52] (Table 1). In contrast to other cyanobacteria that grow exclusively in freshwater bodies, *L. majuscula* has been found to produce other varieties that are just as toxic [66]. In fact, over 70 biologically active components have been identified, of which many are highly toxic. *L. majucula* grows at the bottom of water bodies, usually attached to seaweed and resembles filamentous threads that are approximately 10–30 cm in length [67]. *L. majuscula* was identified back in 1912; however, it was not until the late 1950s that it was reported as the culprit behind an outbreak of acute contact dermatitis. Around 125 individuals swimming in the sea in Oahu, Hawaii, were reported to have acute dermatitis that was later shown to be caused by *L. majuscula* [68]. The symptoms included a burning sensation in the genital and perianal areas that appeared a few hours post-exposure, in addition to skin reddening that developed into blisters and desquamating patches on the skin [68]. During a histopathological examination, the skin displayed rapid onset of vesicular dermatitis and a microscopic examination revealed superficial desquamation, with vesicles ranging in size, in addition to edema of the epidermis, infiltrated by polymorphonuclear leukocytes. Many of the vesicles also contained polymorphonuclear cells, as well as red blood cells, and the dermis layer of the skin revealed inflammatory cell infiltration, such as from eosinophils and neutrophils [68]. Further testing was carried out to ascertain that *L. majuscula* was the antagonist behind the acute dermatitis. This was carried out through patch-testing human volunteers, who all reacted similarly to *L. majuscula* [68]. The reaction to this cyanobacterium and the development of acute dermatitis were observed to follow a certain chronological order. Initially, people come into contact with *L. majuscula* after swimming in water rich in seaweed, which the cyanobacterium grows on. The impact can be exacerbated where individuals continue to wear their swimsuits for a while after leaving the water. After this, the symptoms begin to manifest, including a burning sensation and pruritus. Acute dermatitis occurs 3–8 h after the initial contact [68]. In 1968, beachgoers in Okinawa, Japan, developed acute dermatitis and experienced similar symptoms to those experienced in Hawaii. However, it was not until 1973 that samples were collected from the beach and an unidentified toxin was isolated, which was later shown to be a mixture of debromoaplysiatoxin and aplysiatoxin [69]. These two toxins were initially extracted from sea hare, a marine organism that feeds on filamentous algae. The exposure to a toxin extract from this organism was shown to cause skin-related symptoms. Moreover, DAT and AT were observed to possess robust tumor-promoting functions. The toxins are structurally similar, except for a bromine on the aromatic ring of AT [70]. The absence of a bromine molecule in DAT makes it less capable of inducing cell transformation and subsequent malignancy [71]. Albeit both toxins are capable of causing dermatitis; however, only in vitro results from exposure to the AT toxin were shown to induce a high rate of malignant cell transformation [72].

On the other hand, lyngbyatoxin A structurally resembles an isomer of telocidin A, an indole alkaloid, found in different *Streptomyces* strains. This toxin is known for its potential dermatotoxicity and tumor-promoting function [73].

In addition to the threat that *L. majuscula* poses to human health, previous work in the literature has shown that it also has detrimental environmental impacts that severely harm wildlife. Ref. [68] reported that many horses died after consuming washed-up materials contaminated with *L. majuscula* in Sri Lanka [68]. Furthermore, the tumor-promoting factors in terms of *L. majuscula* have been identified as the causative agent behind a benign, yet detrimental, disease in sea turtles, called fibropapillomatosis, the latter of which manifests in sea turtles after they consume algae that belongs to the *Gracilaria* species, which is usually associated with *L. majuscula*, as it grows on it and derives its nutrients from it without harming it [74]. Other studies have shown that *L. majuscula* can also boost viral synthesis and the malignant transformation of cells [75]. It was also shown to act as an immunosuppressive agent, reducing the proper response of the immune system by antagonizing immune surveillance systems [76].

### 2.2. Anabaenopeptins

Anabaenopeptins are a group of cyclic peptide toxins produced by various cyanobacteria, most notably species from the genus *Anabaena*, as well as *Nostoc* and *Oscillatoria*. *Anabaena* sp. strain 90, found in southern Finland, was shown to produce three distinct anabaenopeptins: A, B, and C [62]. The actions of these toxins have been shown to induce the relaxation of noradrenaline-induced contractions in rats and to inhibit serine proteases; however, their full effects remain underexplored [32]. Structurally, anabaenopeptins are composed of cyclic peptides, often incorporating non-proteinogenic amino acids, which contribute to their stability and bioactivity [77]. Due to their diverse mechanisms of action, anabaenopeptins have been studied for their potential application in medicine, including the development of novel drugs for neurological and infectious diseases. For example, they can inhibit carboxypeptidases [78], protein phosphatases [63], and serine proteases, such as trypsin and chymotrypsin [61]. In aquatic environments, these toxins can accumulate in aquatic food webs, but with unknown consequences for human health [33]. Ongoing research is focused on understanding the full spectrum of toxicity of anabaenopeptins, including their potential carcinogenic and immunotoxic effects. Anabaenopeptins may act as tumor promoters, as some forms are capable of inhibiting protein phosphatase 1 and 2A, similar to MCs and nodularins [78,79,80].

It is important to note that another group of cyclic peptides, known as microginins, are N-acyl lipopeptides found in the genera *Microcystis*, *Nostoc*, and *Planktothrix* (formerly *Oscillatoria*). These compounds are characterized by a beta-amino acid at the N-terminus and C-terminal tyrosine residues [81]. Microginins exert their effects by inhibiting aminopeptidase M, an enzyme involved in angiotensin conversion [82].

### 2.3. Beta-Methylamino-L-Alanine (BMAA)

One of the most prominent links made between toxins produced by *Nostoc* sp. and human neurotoxicity is a disease known as amyotrophic lateral sclerosis/parkinsonism–dementia complex (ALS-PDC) [17]. ALS is a progressive neurodegenerative disease associated with high mortality, as most patients expire due to their symptoms within 3 years of disease onset [83,84,85,86,87]. Sporadic ALS has invariable incidence rates worldwide, except for three regions in the western Pacific, where ALS commonly occurs simultaneously with Parkinson’s disease and Dementia (ALS-PDC). These locations are the Mariana Islands (Guam and Rota), the northern Kii Peninsula in Japan, and the southern lowlands of western New Guinea [88].

The correlation between high rates of ALS-PDC in these regions and cyanobacteria can be elucidated through the diet that the inhabitants of these regions regularly consume. The Chamorro people, who are the indigenous inhabitants of the Marianna Islands, rely majorly on cycad seeds as a source of carbohydrates. These seeds were contaminated with a toxin that was later considered to be the likely etiological agent behind ALS [89]. This theory was rejected multiple times; however, it has resurfaced due to extensive research. Some animal model experiments failed to correlate ALS-PDC with cycad seeds after feeding the material to animals and, as a result, the hypothesis was rejected for the first time [89].

The compound that was hypothesized to be associated with this disease is beta-methylamino-L-alanine (BMAA), which was discovered incidentally, when a group of researchers were studying the seeds of *Lathyrus sativus*. These seeds contained a compound called beta-N-oxalyl-L-diaminopropionic acid (BOAA), which was considered the likely cause of Lathyrism, an upper motorneuron disease in humans [90]. When researchers searched for BOAA in cycad seeds, they discovered the previously unknown compound BMAA [91]. Research into BMAA revealed that it is toxic to both mice and non-human primates; however, only acute manifestations of this compound were observed, which are not associated with chronic ALS-PDC [92]. The potency of both BOAA and BMAA arises from the fact that they are not synthesized from the 20 common amino acids. Instead, they are composed of non-protein amino acids, which are considered toxic, and serve several functions, including protecting plants against predators and storing nitrogen molecules [93]. These findings revived the theory that cycad seeds are the causative agent of ALS-PDC; however, this notion was refuted again when another group of researchers found that cycad seeds contain only low concentrations of BMAA, an insufficient amount to cause the disease when compared to the much higher doses used in animal model experiments [94,95,96,97,98]. This could be attributed to the fact that the Chamorro people wash cycad seeds thoroughly to make sure they are fit for human consumption [89].

Recently, this theory has been revisited after multiple pieces of research have been carried out into cycad BMAA and its relationship with ALS-PDC. It was observed that BMAA in cycad seeds is derived from a certain species of cyanobacteria that resides in the roots of *Cycas micronesica* and have a symbiotic relationship with it [53,99,100,101,102,103,104,105]. Other findings have revealed that BMAA exists in a protein-bound form in cycad flour, rather than a free unbound form, and that it accumulates in the brains of humans with ALS-PDC. This was later confirmed in the work by Murch et al. (2004), who showed that concentrations of protein-bound BMAA were significantly (almost 100-fold) higher in the brains of patients with ALS-PDC than the free form [104,106]. It has been shown that the cyanobacterium belonging to the genus *Nostoc* has a relationship with cycad seeds, providing the seeds with nitrogenous compounds [107]. Furthermore BMAA was shown to play an integral role in cyanobacterial growth, as it assists with iron binding in microorganisms [107]. The Chamorros people have consumed large quantities of BMAA-containing foods, including cycad flour and meat from flying foxes, pigs, and other animals that eat cycad seeds [102]. Interestingly, it was observed that the meat from flying foxes contained high levels of BMAA in the free form and, hence, did not manifest the toxic effects of this compound. This could be due to different reasons, including the prevention of BMAA integration into the flying fox brain through the action of transfer RNA and the fact that flying foxes have a short lifespan, not long enough to display the full effects of BMAA [89].

It was shown that BMAA can be synthesized by both symbiotic and free-living cyanobacteria, hence the chances of human exposure to BMAA are greatly elevated [108]. The incidence of ALS-PDC has been largely attributed to environmental factors. Over the past 5 decades, there has been a notable decline in the incidence of ALS in Guam, which has been shown to be proportional to a decrease in cycad flour consumption and a significant decline in the number of flying foxes due to overhunting [53,103,109,110,111].

There are many parameters to consider when explaining the mechanism of action of BMAA and how it leads to ALS-PDC. It is important to note that glutamate and aspartate are excitatory amino acids that stimulate glutamate receptors and result in a high level of calcium influx into the motor neuron, which ultimately results in cell death [112,113]. Decreased quantities of glutamate transporters appear to play a crucial role in the development of motor neuron death in ALS [114,115]. Studies have shown that patients with ALS have higher plasma concentrations of glutamate when compared to controls. BMAA, a non-protein neurotoxic excitatory amino acid, seems to elicit neurotoxicity by activating glutamate receptors [105]. NMDA receptors are glutamate receptors that constitute the main site of action of BMAA.

BMAA is an NMDA receptor agonist that has been shown to elicit excitotoxicity through the overactivation of this receptor, leading to neuronal death associated with Parkinson’s disease, Alzheimer’s disease, and ALS [116]. For BMAA to activate the NMDA receptor, its beta group must interact with a bicarbonate to form a molecule that can fully activate the receptor [117,118]. Spinal motoneurons appear to be particularly susceptible to the effects of BMAA [105], as BMAA induces the formation of post-synaptic vacuoles, leading to neuron death. Antagonizing the NMDA receptor seems to provide protection against the effects of BMAA [119].

Other mechanisms of action of BMAA have also been theorized. For example, when BMAA is integrated into neurons, conformational changes in neuronal proteins ensue. These misfolded proteins have been implicated and, thus, have been extensively studied in regard to many neurodegenerative diseases [120]. Furthermore, a study conducted by Lobner and colleagues (2007) revealed that in addition to its effects on the NMDA receptors, BMAA is an agonist of the metabotropic glutamate 5 receptor and its effect on this receptor leads to higher levels of oxidative stress [120].

Oxidative stress occurs due to the inhibitory effects of BMAA on the cysteine/glutamate antiporter xc − system, which results in a decrease in the amount of intracellular glutathione. BMAA also functions as a metal ion chelator, forming more oxidative centers and eventually increasing oxidative stress [89]. Several other mechanisms have been proposed to explain the neurotoxicity of BMAA; however, they remain under researched and further investigations are required in order to form a better understanding of such mechanisms and provide clarification.

### 2.4. Guanitoxins (Formerly Anatoxin-A(S))

Another potent and greatly underexplored toxin is anatoxin-a(S), a neurotoxin produced by freshwater cyanobacteria [121]. Unlike, anatoxin-a(S) has a different mechanism of action, in which it acts as an active site-directed irreversible inhibitor of acetylcholinesterase [122,123]. Its molecular structure is also different from that of anatoxin-a and has been identified as an organophosphate [124]. For many years, little was known about this cyanotoxin due to several factors, including fewer reported cases, a difficulty in obtaining the purified compound, and a lack of information about its biosynthesis genes [121]. It has only been isolated from freshwater cyanobacteria, mainly *Dolichospermum* (formerly known as *Anabaena*) and *Sphaerospermopsis* [125]. Additionally, it has been established that anatoxin-a(S) is nearly 10 times more potent than anatoxin-a [121].

### 2.5. Nodularins

Nodularins (NODs) are cyanotoxins produced by *Nodularia spumigena* and *Raphidiopsis* sp. (Table 1). These toxins are structurally similar to MCs and are widely distributed in various environments [126]. Nodularins are cyclic pentapeptides that are characterized by their chemical stability and water solubility, which is attributed to their structure and the presence of several amino acids that are resistant to heat, oxidation, and hydrolysis [127,128]. NODs are predominantly found in brackish waters and are particularly abundant in the Baltic Sea, where they tend to form blooms during the summer months [28,129]. There have been several documented cases of NOD toxicity in animals, including horses, pigs, dogs, and sheep. However, no human incidents have been reported to date [130,131,132,133,134].

Over the past three decades, the effects of NODs on both humans and the environment has garnered increasing attention, with numerous reports emerging from countries such as Australia, Brazil, China, the UK, and the USA [6]. Although the World Health Organization (WHO) has not issued specific guidelines on NOD toxicity, their toxicity is often assessed in relation to MCs, given their structural similarities. Consequently, the same daily intake references that are applied to MCs are also used for NODs [135].

## 3. Cyanotoxins in Aerosols

In addition to the aforementioned routes of exposure, some cyanobacterial species can exist in aerosolized form, with researchers identifying some that are among the least studied taxa of cyanobacteria [136] (Table 1). While research on aerosolized cyanobacteria is still developing, the least studied taxa tend to be those that are not as widely distributed or studied in the context of airborne transmission. Some groups are less frequently examined due to their ecological niches, rarity in aerosol form, or challenges in regard to identification and sampling. Bioaerosols refer to the presence or organisms or their metabolic byproducts in the atmosphere [137]. They are classified as particulate matter, with sizes ranging from 0.2 to 100 μm [136]. These bioaerosols are massively diverse and include a wide range of microorganisms, such as viruses, fungi, microalgae, and cyanobacteria [137]. These aerosolized microorganisms are influenced by several meteorological factors and, consequently, their survival depends on their level of tolerance to different environmental conditions. Such environmental factors include temperature, wind, humidity, and exposure to sun light [138,139]. Once airborne, these microorganisms are exposed to many stressors that threaten their survival, including UV radiation, desiccation, and pollutants [138]. Research has shown that different cyanobacterial species possess varying levels of tolerance to humidity [138]. Another important factor that directly impacts the conditions according to which cyanobacteria can become aerosolized is size; the smaller the microorganism is, the more likely it is to become aerosolized [54]. The presence of cyanobacteria in the atmosphere poses a direct threat to human health and provides a new pathway through which cyanotoxins can be transmitted to humans. Furthermore, the scarcity of literature about this topic constitutes a major obstacle to understanding these microorganisms and accurately determining the gravity of the damage they can cause. Research performed in different locations around the world has revealed that the composition of bioaerosols differs in different areas; however, cyanobacteria were detected in all the samples [140,141]. The findings from recent research suggest that cyanobacteria in the atmosphere can accumulate and act as vectors for the transportation of harmful substances, including pesticides, radionuclides, heavy metals, and some carcinogenic agents, all of which are non-biodegradable and are significantly harmful to humans [54,142].

Another important parameter to consider when discussing bioaerosols is indoor environments where humans spend most of their time, such as homes and workplaces. These spaces have been shown to harbor many types of bioaerosols that may threaten human health [143,144]. These microorganisms can be introduced into indoor environments through different human activities, open windows and doors, and ventilation systems. This is considered a significant route according to which cyanobacteria can cause disease in humans, since people tend to spend the majority of their time indoors [145]. In a study conducted by Bernstein and Safferman (1966), they noted the presence of different species of cyanobacteria in household dust, including *Anabaena* sp. and *Schizothrix* sp. [146].

Exposure to cyanobacteria and their toxins through aerosols can have direct harmful effects on human health, as these microorganisms can be small enough to penetrate through the respiratory tract, all the way to the alveoli, resulting in various types of diseases [147]. Researchers have linked cyanobacteria in aerosols to different health conditions, including allergies, hay fever, rhinitis, respiratory infections, and many others [148]. Lewandowska et al. (2017) revealed that toxic cyanobacteria commonly encountered in the atmosphere are typically no more than 3.3 µm in diameter, which enables these microorganisms to invade the respiratory tract and cause negative effects [54]. Moreover, Facciponte et al. (2018) observed that the upper respiratory tract in humans was frequently targeted by cyanobacteria [55]. These occurrences were consistent in both summer and winter, showing no seasonal pattern. According to scientists from Florida Gulf Coast University, both NODs and MCs were found in the atmosphere, but at low concentrations [149]. The inhalation of MCs can result in harmful effects to different organs, even at lower doses than usual [142]. These cyanotoxins can enter the human respiratory system and can be deposited there as a result of water-based recreational activities that lead to the emission of toxins and subsequent human exposure [24]. Currently, there are many regulations in place to prevent and prohibit swimming in contaminated waters, particularly during toxic algal blooms. This is one way to avoid exposure to cyanotoxins; however, more regulations should be implemented to try and completely abolish exposure to these toxins, as simply avoiding contaminated water is not enough [54,144].

Recently, researchers have been developing HAB prediction models for specific aquatic environments [150]. For example, a group of researchers from South Korea created a HAB predictability model that can forecast the likelihood of a HAB developing based on environmental levels of certain parameters, such as nitrogen, phosphorus, and pH [151]. Other research groups have also focused on developing novel methods to continuously monitor and predict the behavior of HABs, in order to address the public health threats they pose [150].

## 4. Advancements in Detection and Treatment Methods for Cyanotoxins

Cyanotoxins present in water bodies, which are used for irrigation, can work their way up the food chain, contaminating crops consumed by both humans and animals [152]. This highlights the need for effective detection methods, and several new approaches have been developed to identify the presence of cyanotoxins. For example, ref. [153] proposed the use of aptamers, oligonucleotides or peptides that bind to specific targets at varying concentrations, as a sensing technology to detect cyanotoxins.

To reduce or eliminate cyanobacteria and their metabolic byproducts from water sources, various methods have been implemented. Physical and biological methods are effective when treating water bodies, while hybrid and chemical methods are more successful in water treatment plants [150]. Special attention should be given to certain cyanotoxins, such as anatoxin-a and saxitoxins, produced by *Anabaena* and *Aphanizomenon*, which require specific removal strategies [122]. Ozonation and the Fenton process have been proven to be highly effective in degrading anatoxin-a, with the latter achieving total degradation within 90 s [154,155].

Biological methods include the use of cyanophages, viruses that prey on cyanobacteria [150], and algaecide-secreting bacterial species, like *Pseudomonas simiae* OLi, for in situ treatment of harmful algal blooms (HABs) [156]. Physical methods include the use of filtering membranes, commonly employed in water treatment plants, and adsorption with activated carbon, often combined with membrane filtration and pre-oxidation [150,157]. Dredging, which removes nutrient-rich soil layers to limit nutrients for cyanobacteria, is frequently used in lakes and reservoirs [150]. Chemical methods, such as coagulation and chlorination, are also widely used in water treatment plants [150]. Hybrid removal methods, such as coagulation-based removal with UV irradiation [158] and photocatalysis-based hybrid removal, can degrade both cyanobacteria and their toxins [159]. Despite promising advancements in cyanotoxin detection and removal methodologies [160], several challenges remain, particularly in the context of biosensing technologies. Many methods still rely on time-consuming laboratory testing and are location specific, limiting their broader application [150]. Furthermore, current treatment methods often address cyanobacteria and cyanotoxins separately. This approach overlooks the risks of cell-lysing techniques, which can release stored toxins and exacerbate contamination [150]. To address this, integrated strategies that simultaneously target both the microorganisms and their toxins must be developed.

Another limitation is that many studies investigating treatment methods use laboratory-grade water artificially contaminated with cyanobacteria. This setup may not accurately reflect real-world conditions. To improve reliability, treatment methods should be tested in naturally contaminated waters, which better represent the environmental challenges encountered in the field [150].

## 5. Conclusions and Future Research Directions

This review highlights the significant environmental and public health risks posed by various cyanobacterial species and their metabolites, particularly those less commonly studied. Much of the research on cyanobacteria and their toxins has focused on a limited range of toxic species, with *Microcystis* being the primary genus studied. However, recent research indicates that non-*Microcystis* HABs are becoming more common, often exhibiting different and sometimes more aggressive behaviors compared to *Microcystis* HABs. This shift underscores the need for further investigations into a broader array of cyanobacterial species [161]. Key concerns include the potential for human exposure to cyanotoxins, such as MCs and NODs, through ingestion, skin contact, and aerosolized particles, with the latter presenting a growing threat to respiratory health. Additionally, the cardiotoxic effects of MCs, even at low exposure levels, raise serious public health concerns, particularly given that many of the underlying mechanisms remain poorly understood. The role of cyanobacteria in HABs and their impact on ecosystems further emphasizes the urgency of addressing these risks. Notably, there is an emerging link between cyanobacteria, particularly *Lyngbya* species, and neurodegenerative diseases, like ALS, which highlights the need for further research on the neurotoxin BMAA.

While cyanobacterial research has advanced significantly, major gaps remain in understanding their biodiversity, ecological roles, molecular mechanisms, and potential for biotechnological applications. The growing recognition of their importance in biogeochemical cycles and their potential in terms of addressing global challenges, such as climate change and resource sustainability, makes addressing these gaps increasingly urgent. More integrated approaches that combine genomics, ecology, biotechnology, and environmental science are key to overcoming these challenges.

To fill the existing knowledge gaps, future research should prioritize continuous environmental monitoring, multidisciplinary studies on aerosolized cyanobacteria, and investigations into the long-term health impacts of these toxins. Establishing international guidelines on exposure limits for cyanotoxins is also essential for the development of effective public health policy. Overall, a comprehensive approach is necessary to safeguard human health and ecosystems from the threats posed by these microorganisms.

## Figures and Tables

**Table 1:** Different cyanotoxins, their characteristics, and the various hazardous effects on both humans and the environment.

Cyanotoxin	Producing Taxa	Chemical Structure	Toxicity	Mechanism of Action	Environmental Hazard	Treatment/Prevention	Reported Cases	Ref.
Microcystins	*Microcystis* *Dolichospermum (formerly Anabaena)* *Leptolyngbya* *Nostoc* *Synechococcus* *Planktothrix*	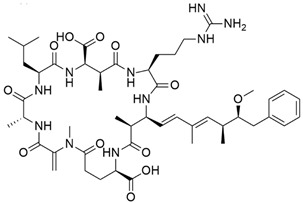 Microcystin from *Microcystis*	Hepatotoxicity Cardiotoxicity	Inhibition of protein phosphatases 1 and 2A, resulting in over phosphorylation	Found in freshwater and can form HABs	No specific antidote Supportive treatment Activated charcoal	Contamination in dialysis units led to the death of 60 patients (Caruaru, Brazil; 1996)	[50,51]
Lyngbyatoxin A	*Lyngbya*	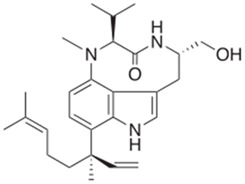	Dermatotoxicity Neurotoxicity	Activation of protein kinase C leading to inflammation and tumor promotion	Contamination of aquatic environments Affects beachgoers	Symptomatic care Avoid exposure/contact	125 cases of severe contact dermatitis in swimmers exposed to *Lyngbya* (Oahu, Hawaii; 1959)	[52]
BMAA	*Anabaena* *Nostoc* *Cylindrospermopsis* *Microcystis* *Chroococcus* *Merismopedia* *Synechocystis* *Myxosarcina* *Leptolyngbya* *Lyngbya* *Oscillatoria*	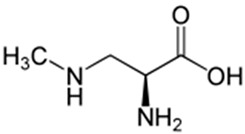	Neurotoxicity	Overstimulation of glutamate receptors	Bioaccumulation in terrestrial and aquatic environments	No specific antidote Contact avoidance Focus on neuro-protective strategies	ALS-PDC in Chamorro people linked to traditional diet (Guam; 1967)	[53]
Airborne cyano-toxins	*Microcystis* *Anabaena* *Nodularia*	Various	Respiratory effects Neurotoxicity Hepatotoxicity	Underexplored; however, exposure via inhalation elicits symptoms	Airborne Can travel long distances Spread through wind and water	Supportive treatment Air filtration Avoid contact	Aerosolized cyanobacteria caused respiratory issues for nearby residents (FL, USA)	[54,55]
Nodularins	*Nodularia spumigena*	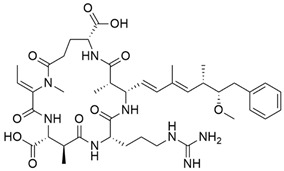	Hepatotoxicity	Inhibition of protein phosphatases 1 and 2A	Brackish waters Bioaccumulate in aquatic environments	Symptomatic treatment	Detected in Baltic Sea fish, potentially hazardous to consumers	[6]
Anatoxins	*Dolichospermum* *Oscillatoria* *Phormidium* *Cuspidothrix* *Aphanizomenon* *Anabaena*	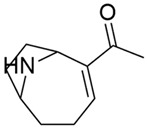 Anatoxin-a	Neurotoxicity	Binds irreversibly to acetylcholine receptors	Freshwater environments Rapid onset of symptoms	No specific antidote Artificial respiration in the case of exposure to anatoxins	Anatoxin-A bloom in contaminated water supply, causing symptoms in residents and affecting over 400,000 people (Toledo, OH, USA; 2014)	[48,56,57]
Anatoxin a(S)	*Anabaena* *Sphaerospermopsis*	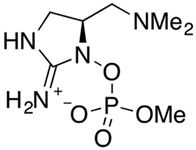	Neurotoxicity	Irreversible inhibitor of acetylcholinesterase	Freshwater environment	Atropine	Sporadic outbreaks in western Canada	[58]
Saxitoxins	*Lyngbya* *Planktothrix* *Aphanizomenon* *Dolichospermum* *Raphidiopsis* *Cylindrospermum*	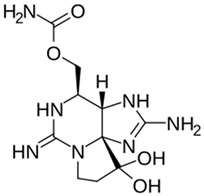	Neurotoxicity	Blockage of voltage-gated Na^+^ channels in neurons	Bioaccumulate in shellfish	CDC recommends Atropine in regard to saxitoxin poisoning Avoid contact	Outbreak of paralytic shellfish poisoning occurred along the US Pacific coast, affecting 107 people who consumed contaminated shellfish (1987)	[57,59,60]
Anabaenopeptins	*Nostoc* *Planktothrix* *Microcystis* *Nodularia* *Anabaena*	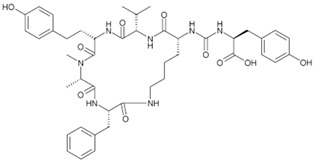 Anabaenopeptin A from *Microcystis aeruginosa*	Neurotoxicity	Inhibitor of carboxy-peptidases, protein phosphatases, and serine proteases	Contamination of lakes and other water bodies	No specific antidote/avoid contact	No well-documented outbreaks	[61,62,63]

## Data Availability

No new data were created or analyzed in this study.
